# New Delhi Metallo-β-Lactamase–Producing *Escherichia coli* Associated with Endoscopic Retrograde Cholangiopancreatography — Illinois, 2013

**Published:** 2014-01-03

Infections with carbapenem-resistant *Enterobacteriaceae* (CRE)* are increasing among patients in medical facilities (1). CRE that produce *Klebsiella pneumoniae* carbapenemase (KPC) have been responsible for much of the increase in the United States. However, New Delhi metallo-β-lactamase (NDM)–producing CRE have the potential to add to this burden. Since first reported in 2009, through 2012, 27 patients with NDM-producing CRE have been confirmed by CDC from isolates submitted by state laboratories. Since January 2013, a total of 69 patients with NDM-producing CRE have been identified in the United States; 44 patients were from northeastern Illinois.

From March to July 2013, nine patients with positive cultures for NDM-producing *Escherichia coli* (eight clinical cultures and one rectal surveillance culture) were identified in northeastern Illinois. An investigation was conducted to understand and prevent the transmission of NDM-producing CRE. A case was defined as an NDM-producing *E. coli* isolate, recovered from a patient in northeastern Illinois, with >85% similarity by pulsed-field gel electrophoresis (PFGE) to the outbreak strain, detected after January 1, 2013. Of the nine cases, eight were treated at the same hospital (hospital A). To determine risk factors for acquiring NDM-producing CRE, a case-control study was conducted. The eight patients cared for at hospital A were selected as case-patients; 27 controls were randomly selected from among 131 hospital A patients with negative surveillance cultures. A history of undergoing endoscopic retrograde cholangiopancreatography (ERCP)† at hospital A was strongly associated with case status (six of eight [75%] versus one of 27 [4%]; odds ratio = 78.0; 95% confidence interval = 6.0 to >999.99).

After manual cleaning and high-level disinfection in an automated endoscope reprocessor, cultures were obtained from the ERCP endoscope used on five of the case-patients. NDM-producing *E. coli* and KPC-producing *K. pneumoniae* were recovered from the terminal section (the elevator channel) of the device.§ The *E. coli* isolate was highly related (>95%) to the outbreak strain by PFGE. Retrospective review and direct observation of endoscope reprocessing did not identify lapses in protocol. Previous studies have shown an association between ERCP endoscopes and transmission of multidrug-resistant bacteria; the design of the ERCP endoscopes might pose a particular challenge for cleaning and disinfection (2,3).

Among 91 ERCP patients who were initiallty notified that they had potential exposure to a culture-positive endoscope, 50 returned for rectal surveillance cultures. NDM-producing *E. coli* were recovered from 23 (46%). An additional 12 patients with NDM-producing CRE have been identified in northeastern Illinois, bringing the total during January–December 2013 to 44. In September 2013, as a result of the investigation, hospital A changed ERCP endoscope reprocessing from automated high-level disinfection to gas sterilization with ethylene oxide; no new cases with exposure to a gas-sterilized ERCP endoscope have been identified.

This investigation highlights the potential for CRE transmission following ERCP. Health-care facilities with CRE outbreaks should consider the possibility of ERCP-related transmission. If ERCP-related transmission of CRE is suspected, reprocessing and preventative maintenance procedures for ERCP endoscopes should be evaluated in consultation with the manufacturer of the endoscope and automated endoscope reprocessor, if used. In addition, expertise in the evaluation and prevention of CRE transmission are available at CDC and can be accessed via state and local health departments.

## Reported by

*Mabel Frias, MPH, Cook County Dept of Health; Victoria Tsai, MPH, Illinois Dept of Public Health. Heather Moulton-Meissner, PhD, Johannetsy Avillan, PhD, Div of Healthcare Quality Promotion; Lauren Epstein, MD, Jennifer Hunter, DrPH, M. Allison Arwady, MD, EIS officers, CDC.* ***Corresponding contributor:*** *Lauren Epstein, xdd0@cdc.gov, 404-639-8162.*

## Footnotes

* Additional information about CRE is available at http://www.cdc.gov/hai/organisms/cre/cre-patientgeneral.html.

† An endoscopic procedure used to diagnose and treat problems of the bile and pancreatic ducts.

§ An illustration identifying the parts of the elevator channel is available at http://www.health.qld.gov.au/endoscopereprocessing/flash/432.asp.
